# Maximum likelihood phylogeographic inference of cell motility and cell division from spatial lineage tracing data

**DOI:** 10.1093/bioinformatics/btae221

**Published:** 2024-06-28

**Authors:** Uyen Mai, Gary Hu, Benjamin J Raphael

**Affiliations:** Department of Computer Science, Princeton University, 35 Olden Street, Princeton, NJ 08540, USA; Department of Computer Science, Princeton University, 35 Olden Street, Princeton, NJ 08540, USA; Department of Computer Science, Princeton University, 35 Olden Street, Princeton, NJ 08540, USA

## Abstract

**Motivation:**

Recently developed spatial lineage tracing technologies induce somatic mutations at specific genomic loci in a population of growing cells and then measure these mutations in the sampled cells along with the physical locations of the cells. These technologies enable high-throughput studies of developmental processes over space and time. However, these applications rely on accurate reconstruction of a *spatial cell lineage tree* describing both past cell divisions and cell locations. Spatial lineage trees are related to phylogeographic models that have been well-studied in the phylogenetics literature. We demonstrate that standard phylogeographic models based on Brownian motion are inadequate to describe the spatial symmetric displacement (SD) of cells during cell division.

**Results:**

We introduce a new model—the SD model for cell motility that includes symmetric displacements of daughter cells from the parental cell followed by independent diffusion of daughter cells. We show that this model more accurately describes the locations of cells in a real spatial lineage tracing of mouse embryonic stem cells. Combining the spatial SD model with an evolutionary model of DNA mutations, we obtain a phylogeographic model for spatial lineage tracing. Using this model, we devise a maximum likelihood framework—MOLLUSC (Maximum Likelihood Estimation Of Lineage and Location Using Single-Cell Spatial Lineage tracing Data)—to co-estimate time-resolved branch lengths, spatial diffusion rate, and mutation rate. On both simulated and real data, we show that MOLLUSC accurately estimates all parameters. In contrast, the Brownian motion model overestimates spatial diffusion rate in all test cases. In addition, the inclusion of spatial information improves accuracy of branch length estimation compared to sequence data alone. On real data, we show that spatial information has more signal than sequence data for branch length estimation, suggesting augmenting lineage tracing technologies with spatial information is useful to overcome the limitations of genome-editing in developmental systems.

**Availability and Implementation:**

The python implementation of MOLLUSC is available at https://github.com/raphael-group/MOLLUSC.

## 1 Introduction

Development of multicellular organisms is a spatiotemporal process involving growth, death, differentiation, and movement of cells. Lineage tracing, or inferring the complete history of cell divisions during the development of an organism or tissue, has been a key goal of developmental biology. In most organisms—including humans—the rate of naturally occurring somatic mutations is too low to provide sufficient phylogenetic signal to reconstruct a phylogenetic tree from single cells (Chen *et al.*, 2022). There has been tremendous interest in dynamic lineage tracing technologies, which use genome-editing technologies such as CRISPR/Cas9 to induce heritable mutations at pre-defined locations in the genome which are then measured *via* single-cell sequencing of the cells (McKenna *et al.*, 2016, Kalhor *et al.*, 2018, Raj *et al.*, 2018, Spanjaard *et al.*, 2018, Wagner *et al.*, 2018, Chan *et al.*, 2019, Bowling *et al.*, 2020). Multiple specialized computational methods have been developed to infer cell lineage trees from dynamic lineage tracing data (Jones *et al.*, 2020, Feng *et al.*, 2021, Chen *et al.*, 2022, Gong *et al.*, 2022, Seidel and Stadler, 2022, Sashittal *et al.*, 2023, Mai *et al.*, 2024). Some methods also use expression data in addition to the CRISPR-induced mutations (Zafar *et al.*, 2020, Pan *et al.*, 2023). Recently, spatial lineage tracing technologies have emerged that enable recording of spatial location of the cells in addition to their induced mutations (Chow *et al.*, 2021, He *et al.*, 2022, Chadly *et al.*, 2024). Each of these published lineage tracing technologies offers unique combinations of spatial recording and lineage tracking technologies. This raises the question of how to infer a *spatial cell lineage tree*, a tree that records the history of both cell divisions and cell movements through space, from this data.

The inference of a spatial lineage tree is related to questions studied in phylogeography over the past several decades (Lemey *et al.*, 2009, 2010, Nielsen and Beaumont, 2009, Bloomquist *et al.*, 2010, Kalkauskas *et al.*, 2021). A phylogeographic model describes both the evolutionary history and migration history of the species from the observed data of the extant species. In addition, from the inferred phylogeny and spatial distributions of the species, phylogeography studies can answer questions related to the interaction between genetic evolution and spatial migration.

Existing phylogeographic models, however, rely on assumptions that do not always hold in spatial lineage tracing data. For example, nearly all existing phylogeographic models are based on reversible Markov processes that allow for efficient computation (Lemmon and Lemmon, 2008, Lemey *et al.*, 2009). This is a reasonable assumption when there is a notable separation time between parent and child nodes (e.g. hundreds to thousands of generations), and thus directional biases in movement during one generation can be ignored. In contrast, the time scale in spatial lineage tracing is much shorter.

We find that existing phylogeographic models ignore a key property in real spatial lineage tracing data, namely that when a cell divides there is a *symmetric displacement* of daughter cells relative to the parent. We find strong evidence for such symmetric displacement in the intMEMOIR spatial lineage tracing data of mouse embryonic stem cells (Chow *et al.*, 2021).

We propose the symmetric displacement (SD) model to describe the spatial location of daughter cells relative to the parent cell during cell division. To the best of our knowledge, this is the first attempt to model the displacement of cells during cell division in the context of lineage tracing. Combining the SD spatial model with the probabilistic mixed-type missing (PMM) model (Mai *et al.*, 2024) for sequence data, we obtain a phylogeographic model for spatial lineage tracing. We derive a maximum likelihood (ML) method, MOLLUSC (Maximum Likelihood Estimation Of Lineage and Location Using Single-Cell Spatial Lineage tracing Data), that infers a *spatial lineage tree* from spatial lineage tracing data. The inferred spatial lineage tree contains both lineage and spatial information, including the time-resolved branch lengths, spatial diffusion rate, and sequence mutation rate. We show on simulated data that MOLLUSC has higher accuracy in estimation of branch lengths and mutation rate compared to solely using sequence data and accurately estimates the spatial diffusion rate. Applying MOLLUSC to intMEMOIR—a spatial lineage tracing dataset of mouse embryonic stem cells—we detect a clear correlation between cell radius and division displacement.

## 2 Materials and methods

### 2.1 Spatial lineage tracing data and representation of cell lineage tree

Spatial lineage tracing data consists of two modalities: the *observed* CRISPR-induced sequences (i.e. character matrix)—which we denote by **S**—and the spatial locations (i.e. (*x*, *y*) coordinates) of cells existing at the end of the experiment—which we denote by **L**. Because these cells have been divided from a common ancestor (i.e. the *progenitor cell*), they form leaf nodes of a hidden phylogenetic tree of the cells, which we will refer to as the *cell lineage tree*.

The cell lineage tree is a rooted tree $T=(V_{T},E_{T})$ whose branch lengths measure time between consecutive cell divisions. We let $L_{T}$ denote the *set of leaves* of *T*. Let $(u,v)\in E_{T}$ be the edge from *u* to its child *v* (where $u,v\in V_{T}$), and let *r_T_* denote the root of *T*. We will use “edge” and “branch” interchangeably. We assume that the root of *T* has exactly one child (the progenitor cell needs time to divide) and all other internal nodes of *T* have exactly two children (cells always divide into two). When the context is clear, the subscript *T* is omitted for brevity. Let δ(·,·) denotes the distance of two nodes in *T*. For any edge $(u,v)\in E_{T}$, we let *δ_v_* be the shorthand for δ(u,v).

### 2.2 Phylogeographic model

A typical phylogeographic model consists of two independent processes occurring on a lineage tree: (1) sequence evolution and (2) spatial diffusion. The joint likelihood of the sequence data **S** and cell locations **L** given *T* is the product of the two independent likelihoods:

(1) $$\begin{array}{c} L(T,\Omega ;L,S)=L_{L}(T,\Omega_{L},\{\delta_{v}\};L)L_{S}(T,\Omega_{S},\{\delta_{v}\};S), \end{array}$$

where $\left\{\delta_{v}\right\}$ denote the set of branch lengths of *T*, $\Omega_{S}$ and $\Omega_{D}$ denote the parameters of the spatial model and the sequence evolution model, respectively, and $\Omega =(\Omega_{S},\Omega_{D},\{\delta_{v}\})$ is the set of all parameters of the phylogeographic model.

### 2.3 Brownian motion model for spatial diffusion

**Figure 1.**
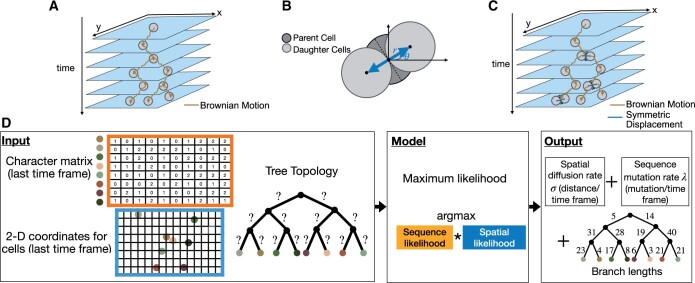
Overview of the symmetric displacement (SD) model and MOLLUSC. (A) The traditional Brownian motion phylogeographic model, where an organism moves according random drift. (B) The center position of two daughter cells (light gray) are placed symmetrically at distance *r* from the center position of the parent cell following cell division. (C) The symmetric displacement (SD) model for spatial lineage tracing data. The spatial locations of cells are a superposition of the initial symmetric displacement of daughter cells from the parent cell followed by Brownian motion. (D) MOLLUSC computes a maximum likelihood phylogeography for spatial lineage tracing data using a joint likelihood of the sequences and the 2D spatial locations.

Consider a cell phylogeny *T* that has $|L_{T}|=N$ leaf nodes. For a node *v* in V(T), let *x_v_* and *y_v_* denote the *x* and *y* coordinates of *v*. We assume that *x_v_* and *y_v_* are given (i.e. observed) for all leaf nodes $v\in L_{T}$ and are hidden for all other nodes. Let $x_{r_{T}}=x_{0},\;y_{r_{T}}=y_{0},\;L_{x}=\{x_{u}:u\in L_{T}\},\;L_{y}=\{y_{u}:u\in L_{T}\}$, and $L=(L_{x},L_{y})$ be the *observed spatial data*. Assuming the spatial diffusion on *x* and *y* coordinates are independent, for all $(u,v)\in E_{T}$ where *u* is the parent of *v*, the Brownian motion model (Lemmon and Lemmon, 2008) assumes that

(2) $$\begin{array}{c} x_{v}=x_{u}\;+\;N(0,\sigma^{2}\delta_{v}),\\ y_{v}=y_{u}\;+\;N(0,\sigma^{2}\delta_{w}), \end{array}$$

where $N(0,\sigma^{2}\delta_{v})$ denotes a Gaussian distribution of mean 0 and variance $\sigma^{2}\delta_{v}$, and *σ* is the diffusion rate. The model is illustrated in Fig. 1A.

A fundamental assumption of the Brownian motion model is the independence of the spatial location of the two daughter nodes given the location of the parent node. While this is a common assumption in a most research works in phylogeography, our analysis of the spatial lineage tracing data from the intMEMOIR technology (see section “Evidence of SD of cell division in intMEMOIR data” in Results) revealed that this assumption does not hold for the positions of dividing cells. Specifically, we detect a non-negligible displacements of the daughter cells from their parent *right after* cell division. Importantly, the displacements are symmetric, breaking the assumption about the independent locations of daughter cells given the parent.

### 2.4 SD model

We introduce the SD model to describe the spatial location of cells over a developmental process. The SD model is a composite of initial displacement by symmetric cell division followed by cell diffusion (movement by Brownian motion). Specifically, for a fixed cell radius *r* and a triplet (*u*, *v*, *w*) where *u* is the parent of *v* and *w*, we assume:

(3) $$\begin{array}{c} x_{v}=x_{u}\;+\;r\;\text{cos}(\theta_{u})\;+\;N(0,\sigma^{2}\delta_{v}),\\ x_{w}=x_{u}\;-\;r\;\text{cos}(\theta_{u})\;+\;N(0,\sigma^{2}\delta_{w}),\\ y_{v}=y_{u}\;+\;r\;\text{sin}(\theta_{u})\;+\;N(0,\sigma^{2}\delta_{v}),\\ y_{w}=y_{u}\;-\;r\;\text{sin}(\theta_{u})\;+\;N(0,\sigma^{2}\delta_{w}), \end{array}$$

where *σ* is the diffusion rate (i.e. $\sigma^{2}$ is the variance of the Gaussian model governing the Brownian motion), and $\left(r,\theta_{u}\right)$ are the polar coordinates of the daughter cells from the parent cell (Fig. 1B) reflecting SD at the division time.

The SD model generalizes existing models in the phylogeography and cell motility literature (Hall, 1977, Pérez and Prendergast, 2007, Codling *et al.*, 2008, Jones *et al.*, 2015, Wadkin *et al.*, 2018). When *r *=* *0, the model reduces to the Brownian motion model, since $\left\{\theta_{u}\right\}$ has no effect on spatial locations. Note that the SD model has one parameter *θ_u_* for each internal vertex and one parameter *r* for cell radius. This model has more parameters than the Brownian motion model and therefore, requires a larger amount of data for parameter estimation. The SD model also has some connection to the *Arithmetic Brownian Motion* (ABM) model (Royer-Carenzi and Didier, 2016), which assumes a continuous character evolves according to Brownian motion with some linear deterministic trend (i.e. given a successive time step of length *s*, the increment can be modeled as $N(\mu s,\sigma^{2}s)$ for a linear constant trend *μ*). Our model differs as in our case we have a one time event (cell division) that does not scale with time between division times, as well as more importantly the symmetric nature of related daughter cells.

### 2.5 Likelihood computation under the SD model

Let $\Omega_{L}=(\sigma ,r,\{\theta_{u}\})$ be the set of all parameters of the SD model. Let $p(L_{x};T,\Omega_{L},\{\delta_{v}\})$ denote the likelihood of $L_{x}$ given $T,\Omega_{S}$ and $\left\{\delta_{v}\right\}$ under the SD model, and similarly define $p(L_{y};T,\Omega_{L},\{\delta_{v}\})$. Assuming the diffusion processes on the *x* and *y* coordinates are independent, we have:

(4) $$L_{L}(T,\Omega_{L},\{\delta_{v}\};L)=p(L_{x};T,\Omega_{L},\{\delta_{v}\})p(L_{y};T,\Omega_{L},\{\delta_{v}\}).$$

From Equation (3), we prove in Supplement that for all leaf nodes *w* and all pairs of leaf nodes v≠w:

(5) $$\begin{array}{cc} & x_{w}∼N\left(x_{0}\;+\;\sum_{\left(u,v)\in \text{Path}(r_{T},w\right)}s_{u,v}r\;\text{cos}(\theta_{u}),\sigma^{2}\delta (r_{T},w)\right),\\ & \text{cov}(x_{v},x_{w})=\sigma^{2}\delta (r_{T},\text{lca}(v,w)). \end{array}$$

Here, Path(·,·) denotes the path between two nodes, lca(·,·) denotes the least common ancestor of two nodes, and $s_{u,v}=1$ if *v* is the left child of *u* and $s_{u,v}=-1$ otherwise. Therefore, the distribution of $L_{x}$ is multivariate normal, with mean $\mu_{x}$ and covariance Σ, where

(6) $$\mu_{x}(w)=x_{0}\;+\;\sum_{\left(u,v)\in \text{Path}(r_{T},w\right)}s_{u,v}r\;\text{cos}(\theta_{u}),\;\forall w\in L_{T},$$

(7) $$\Sigma (w,w)=\sigma^{2}\delta (r_{T},w),\;\text{for all leaf nodes}\;w,$$

(8) $$\Sigma (v,w)=\sigma^{2}\delta (r_{T},\text{lca}(v,w)),\;\text{for all pairs of non-root nodes}\;v\neq w.$$

Similarly for the *y*-coordinate, $L_{y}$ is multivariate normal with $\mu_{y}$ and covariance Σ, where $\mu_{y}$ is defined similarly. Thus,

(9) $$\begin{array}{cc} & p(L_{x};T,\Omega_{L},\{\delta_{v}\})\\ & =\frac{1}{{\left(2\pi \right)}^{N/2}\text{det}{\left(\Sigma \right)}^{1/2}}\text{exp}\;\left[-\frac{1}{2}{\left(L_{x}\;-\;\mu_{x}\right)}^{T}\Sigma^{-1}(L_{x}\;-\;\mu_{x})\right] \end{array}$$

(10) $$\begin{array}{cc} & p(L_{y};T,\Omega_{L},\{\delta_{v}\})\\ & =\frac{1}{{\left(2\pi \right)}^{N/2}\text{det}{\left(\Sigma \right)}^{1/2}}\text{exp}\;\left[-\frac{1}{2}{\left(L_{y}\;-\;\mu_{y}\right)}^{T}\Sigma^{-1}(L_{y}\;-\;\mu_{y})\right] \end{array}$$

We show that Equations (9) and (10) can be computed efficiently by a generalization of the Felsenstein’s algorithm for continuous traits (Felsenstein, 1973). Details are in Supplementary Section S4.

### 2.6 The evolutionary model of CRISPR-induced sequences

We model the evolution of the characters using the PMM model (Mai *et al.*, 2024), which is a continuous-time Markov model specifically designed for CRISPR-induced sequences. The PMM model captures the irreversibility and non-modifiability of the CRISPR-induced mutations, as well as enables the inference of time-resolved branch lengths from sequence data. Below we give a summary of the PMM model. Refer to Mai *et al.* (2024) for more details.

The PMM model is parameterized by the tree branch lengths $\left\{\delta_{v}\right\}$, mutation rate *λ*, heritable missing rate *ν*, and dropout rate ϕ. Let $\Omega_{S}=(\lambda ,\phi ,\nu )$. We refer to the data obtained from the CRISPR-induced sequences as the *observed character matrix*, denoted by **S**, which is an *N *×* K* matrix where *K* is the number of target sites(following the convention in phylogenetics, we use *target site* and *site* interchangeably.) and *N* is the number of cells. Entries in column *k* of **S** take values in the set $A^{\left(k\right)}=\{?,\;-\;1,0,1,\ldots ,M^{\left(k\right)}\}$, where (a) $A^{\left(k\right)}$ is the *alphabet* of target site *k*, (b) 0,−1,? represent the *unmutated state, silent state*, and *missing state*, respectively, and (c) $1,\ldots ,M^{\left(k\right)}$ are *mutated states*. Under the PMM model, the likelihood of each site *k* of **S** is:

(11) $$\begin{array}{cc} & L_{S}(T,\Omega_{S},\{\delta_{v}\};S^{\left(k\right)})\\ & =P(S^{\left(k\right)};T,\Omega_{S},\{\delta_{v}\})\\ & =\sum_{x}P(S^{\left(k\right)},x;T,\Omega_{S},\{\delta_{v}\})\\ & =\sum_{x}\prod_{e=(u,v)\in E_{T}}\Psi_{e}^{\left(k\right)}(x(u),x(v))\prod_{w\in L_{T}}\Phi (x(w),S^{\left(k\right)}(w)), \end{array}$$

where **x** denotes a realization of the ancestral sequences, $E_{T}$ and $L_{T}$ denote the edge and leaf sets of *T*, respectively, Ψ and Φ are the transition probability matrix and the dropout matrix, respectively, as defined in Mai *et al.* (2024). The sequence likelihood, $L_{S}(T,\Omega_{S},\{\delta_{v}\};S)$, is the product of the likelihoods of the individual sites:

(12) $$L_{S}(T,\Omega_{S},\{\delta_{v}\};S)=\prod_{k=1}^{K}P(S^{\left(k\right)};T,\Omega_{S},\{\delta_{v}\}).$$

### 2.7 MOLLUSC: a maximum likelihood method to infer spatial cell lineage tree

Recall that the joint likelihood L(T,Ω;L,S) of **L** and **S** is simply the product of $L_{L}(T,\Omega_{L},\{\delta_{v}\};L)$ and $L_{S}(T,\Omega_{S},\{\delta_{v}\};S)$ (Equation (1)). We infer the maximum likelihood tree from **L** and **S** by solving the following constrained optimization problem:

(13) $$\underset{T,\Omega }{\max}\;\text{log}\;L(T,\Omega ;L,S)$$

such that

(14) $$\begin{array}{c} \sum_{\text{Path}(r_{T},v)}\delta_{v}=\tau_{\text{end}},\;\;\;\text{for all}\;\;\;v\in L_{T}, \end{array}$$

where $\tau_{\text{end}}$ is the length of the experiment (i.e. all cells at the leaves of the lineage tree were sampled at the same time, so they must have the same distance to the root in time unit).

## 3 Results

We provide evidence that the SD of cells can be observed in real data and that it has a noticeable effect on estimating the separation time between parent and daughter cells. Using this evidence as motivation, we benchmark MOLLUSC on (1) different simulation setups and (2) real data from intMEMOIR of dynamic lineage tracing sequences and cell locations, to illustrate the benefit of using the new spatial model on inferring time-resolved branch lengths and the spatial diffusion rate of the cells.

### 3.1 Evidence of SD of cell division in intMEMOIR data

intMEMOIR (Chow *et al.*, 2021) is a recent experimental technology for single-cell spatial lineage tracing that involves a combination of inducible inheritable barcode edits with an imaging system for spatial resolution (refer Supplementary Section S1 for more details). One unique feature of this imaging technology is that ground truth lineage trees and ancestral locations are available. We analyze cell locations from consecutive time frames of the intMEMOIR data *right before and after cell divisions*. In particular, for every time frame *t* that contains a parent cell *p* that divides in the next time frame (*t *+* *1) into daughter cells *c*_1_ and *c*_2_, we and compare the locations of the daughter cells *c*_1_ and *c*_2_ with the location of *p*. From our analysis, we observe that there are *displacements of the cells because of division*, which is distinct from the spatial diffusion of the cells before division that is usually modeled by Brownian motion in phylogeography. More importantly, we also show evidence for the SDs of the daughter cells from their parent.

**Figure 2.**
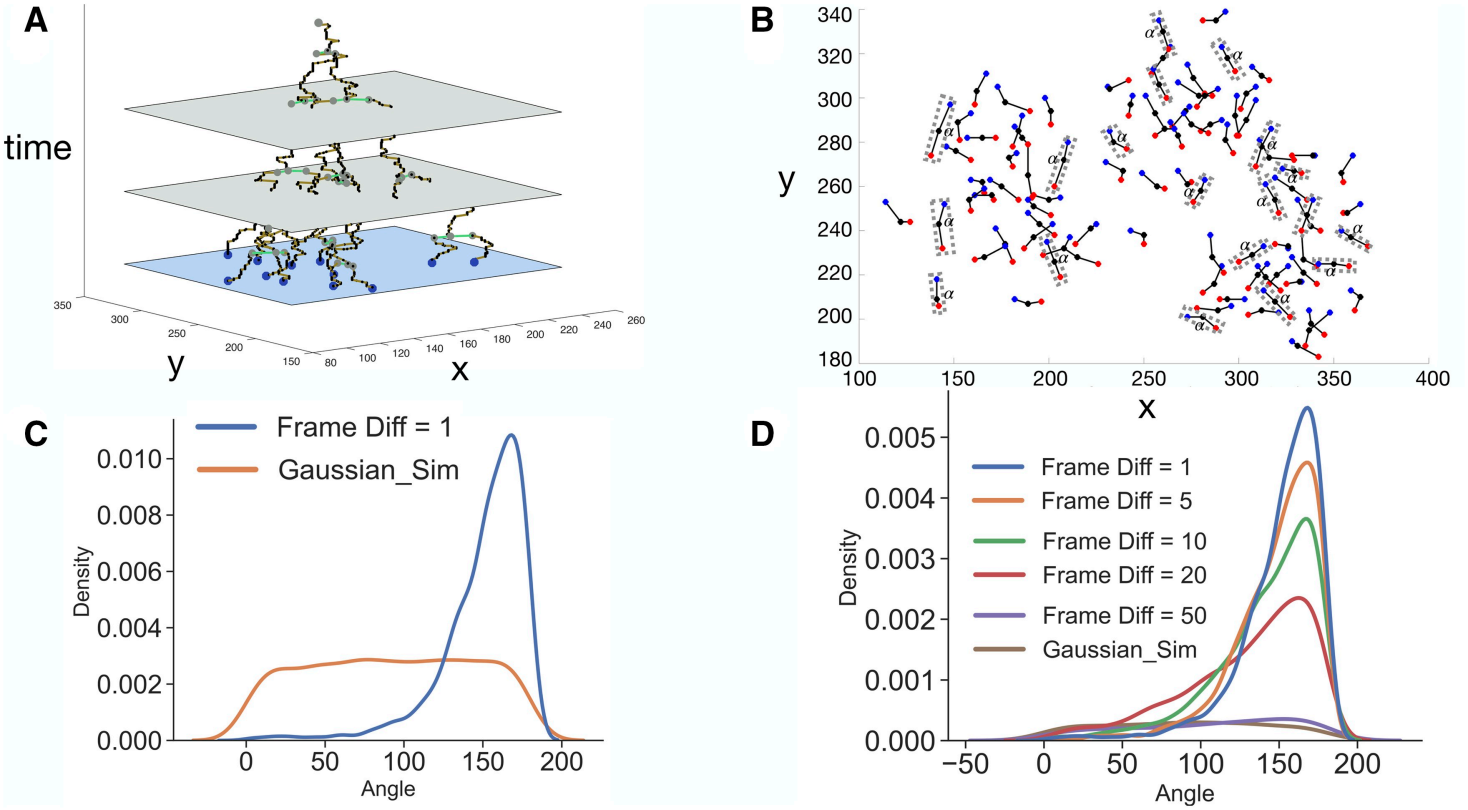
(A) The lineage tree with cell locations of the sample s10c1 of intMEMOIR. Although cell locations are given for all time frames, sequences are only recorded at the last time frame (i.e. Frame 216). In this work, we only use cell locations at the last time frame where sequence data is available. (B) Empirical locations of the parent cell (shown in black) and the two daughter cells (shown in blue and red) at the division frames of the intMEMOIR data (combined of all cells of slide 10). Each gray box indicates a triplet of one parent cell and its two daughters, added for visual purpose. A majority of daughter–parent–daughter angles (*α*) is at high degree (close to 180°) in agreement with the symmetric displacement (SD) model. See Supplementary Fig. S6 for visualization of some other slides of the intMEMOIR data. (C) Empirical distribution of the daughter–parent–daughter angles (*α*) at one time frame after cell division versus the theoretical distribution under the Brownian motion model. The empirical distribution is concentrated at a high value (close to 180°) indicating a symmetric displacement of the two daughter cells from their parent, while the Brownian motion model yields a uniform distribution of the angles. (D) Empirical distributions of the daughter–parent–daughter angles (*α*) after different number of time frames since the division frame. As the time from cell division increases the distribution of the angles approaches the uniform distribution consistent with the Brownian motion model.

We first visualize the locations of each triplet of cells (i.e. the 2D coordinates of the parent cell *p* at time frame *t* and those of its daughters *c*_1_ and *c*_2_ at time frame *t *+* *1) (Fig. 2B). We observe that a majority of these triplets have the daughter cells symmetric from the parent cell. It is non-trivial, however, to either accept or reject the hypothesis of SD by division from such a discrete set of time frames. Note that the displacement is defined as the cell movement *right after the moment of division*. The discretized time frames, however, cannot fully capture this phenomenon, as we only know that cell division happened *some time between* the two time frames that are recorded, but cannot be certain about *the exact moment* of cell division. As such, there can be small diffusion of the daughter cells between the actual time of cell division and the time their locations were recorded, distorting the analysis on SD.

To test our hypothesis about the symmetry of cell displacements versus a sole Brownian motion displacement of the cells, we conducted a more in-depth analysis. We analyze the angles formed from the daughter cells and the parent cell. We name such an angle the daughter–parent–daughter angles, and denote it by α(τ), where *τ* is the time between the recording times of the parent cells and the two daughters. The hypothesis about SD implies that when τ→0, the angle approaches $180^{{}^{\circ}}$. However, because the resolution of recording only allows a minimum *τ* = 1, there is a distribution of *α* among the collected triplets. Nevertheless, from Fig. 2B which shows *α* at *τ* = 1, we can observe that *α* is usually large (close to $180^{{}^{\circ}}$).

In contrast, if there is no displacement by division and cells only move according to Brownian motion, then the daughter cells can form any angle around the parent cell with the same probability, so *α* is uniformly distributed in $\left[0^{{}^{\circ}}\;-\;180^{{}^{\circ}}\right]$. We show that the empirical distribution of *α* at *τ* = 1 is very different from that of the Brownian motion model, with a much higher concentration at the large degree closer to $180^{{}^{\circ}}$ (Fig. 2C, where data are collected for all slides of intMEMOIR). This fact allows us to reject the Brownian motion model at *τ* = 1 and supports the hypothesis of SD right after cell division.

We further study cell movement after division at longer time intervals. By analyzing the distribution of α(τ) at τ > 1, we test for the following two hypotheses: (1) the daughter cells continue moving in the direction defined by the initial displacement from their parent, and (2) each daughter cell diffuses in space independently of the initial direction of the displacement from their parent. Note that if (1) holds, then the daughter cells always maintain symmetric positions around the parent cell, so α(τ) should be large. In contrast, if (2) holds, the angle can change to any value after division time and the distribution approaches a uniform distribution when τ→∞. By constructing the empirical distributions of *α* at different *τ* (Fig. 2D), we observe that the distribution of *α* gradually converges to the uniform distribution with increasing *τ*, and at *τ *= 50, which is the maximum observed branch length of the lineage trees in the intMEMOIR data, we see that the distribution of *α* is very close to the uniform distribution possessing by the Brownian motion model. This result supports the application of the Brownian motion model independently for each daughter cell’s diffusion beyond their initial displacements from the parent cell.

### 3.2 Evaluation of MOLLUSC

We evaluate MOLLUSC on both simulated data and real intMEMOIR data.

#### 3.2.1 Evaluation on simulated data

Using the proposed phylogeographic model, we simulated both sequence and location data of the cells. Both sequences and cell locations were simulated from the real lineage tree topologies (*T*) and real branch lengths $\left\{\delta_{v}\right\}$ of intMEMOIR. We filtered out the lineage trees that have less than 10 leaves, leaving us with 70 model trees used for simulation. For each of these model trees, we simulated spatial data following the SD model and sequence data following the PMM model. The parameters $\Omega_{L}$ of the SD model and $\Omega_{S}$ of the PMM model were selected to match statistics of multiple data modules of intMEMOIR: the frame-by-frame data, the imaging data, and the sequence data (see Supplementary Section S2 for more details). We set the parameters in the simulation as follows: diffusion rate σ=1.5, cell radius *r *=* *6.68, number of target sites *K *=* *10, alphabet size $A^{\left(k\right)}=\{0,1,2\}$ for every site *k*, and mutation rate λ=0.006. In addition, we simulated two other sets of spatial data that have σ=0.5 and *σ* = 3 (other parameters were kept the same). These two additional datasets are useful in studying the effect of different diffusion rates on the performance of MOLLUSC.

We ran MOLLUSC on each of the following two scenarios: (i) fully simulated data: *simulated sequence data* and *simulated location data* were used as inputs to MOLLUSC, and **(**ii) semi-simulated data: *simulated sequence data* and *real location data* were used as inputs to MOLLUSC.

#### 3.2.2 Fully simulated data

**Figure 3.**
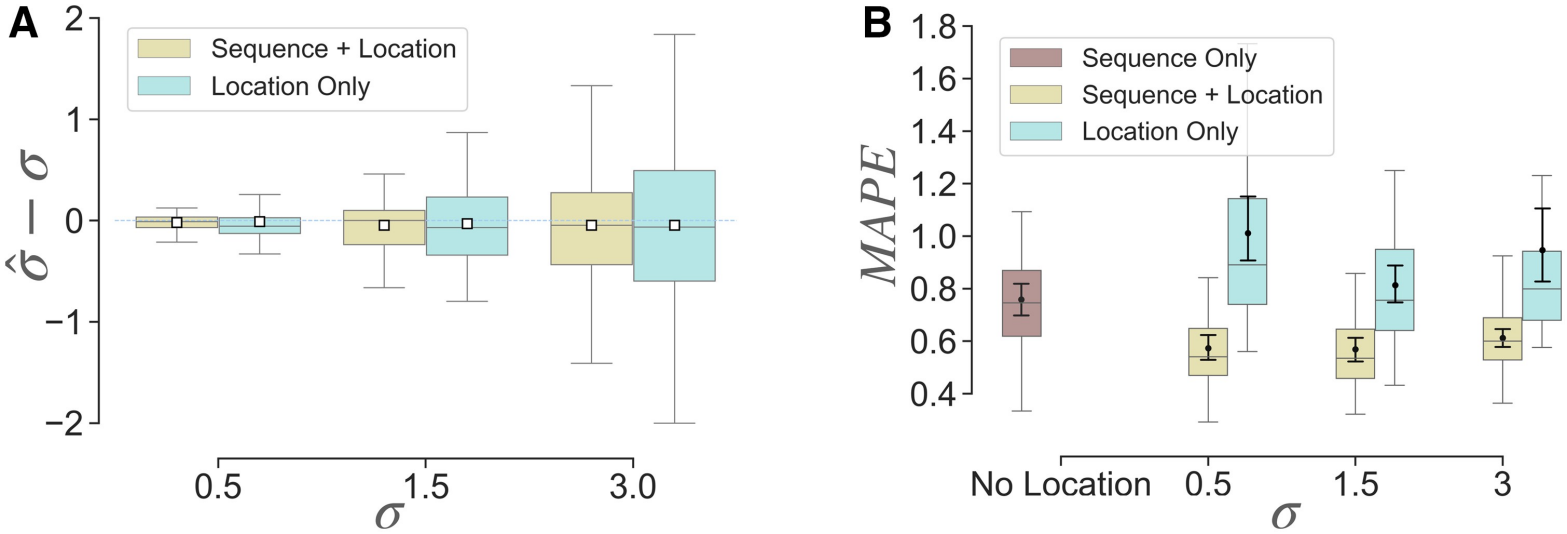
Evaluation on spatial diffusion and branch length estimation error on simulated sequence and location data. Each point represents the result on one tree (70 trees total). (A) Difference $\hat{\sigma }-\sigma$ between the estimated spatial diffusion rate $\hat{\sigma }$ and the true rate *σ* using both sequence and location data, and using only location data. (B) Branch length error measured as the mean absolute percentage error (MAPE) for each tree with simulated sequences and simulated location data.

When running with simulated sequence and simulated location data, the estimate $\hat{\sigma }$ of MOLLUSC is unbiased on all model conditions that we tested (Fig. 3A). The variance of $\hat{\sigma }$ is smaller when both sequence and location data are given than when only location data is given, indicating the benefit of using both data modules for estimation. Interesting but not out of expectation, the variance of $\hat{\sigma }$ also increases with the true value of *σ*, indicating that when cell motility increases, there is more uncertainty in estimation of the diffusion rate.

Next, we study branch length estimation error (Fig. 3C). We measure branch length error by the mean absolute percentage error (MAPE), defined over the set of *n* branches $\left\{\delta_{i}\right\}$ in a given tree and the estimates of those branch lengths $\left\{{\hat{\delta }}_{i}\right\}$ as:

(15) $$\text{MAPE}(\{\delta_{i}\},\{{\hat{\delta }}_{i}\})=\frac{1}{n}\sum_{i=1}^{n}\left|\frac{{\hat{\delta }}_{i}\;-\;\delta_{i}}{\delta_{i}}\right|$$

If only location data is given, branch length error is smallest at σ=1.5, which is the diffusion rate that best matches the real intMEMOIR data. The error increases on both the lower (i.e. σ=0.5) and higher, that is, *σ *= 3 values of *σ*. Interestingly, branch length error of Location Only (L) at σ=1.5 is very similar to that of Sequence Only (S), indicating that the sequence and location data have similar amount of phylogenetic signals (i.e. the average MAPE of Sequence Only is 0.758 and of Location Only at σ=1.5 is 0.813), and the difference is insignificant according to ANOVA test (*P*-value .256). Importantly, employing both sequence and location data significantly improves branch length estimation, where the average error reduces from ≈0.758 in S to ≈0.569 in S + L. The difference between S + L and S is significant, according to ANOVA test (*P*-value $2.8\;\times \;10^{\;-\;6}$). Additionally, the error does not change much with *σ* if both sequence and location data are used.

#### 3.2.3 Semi-simulated data

**Figure 4.**
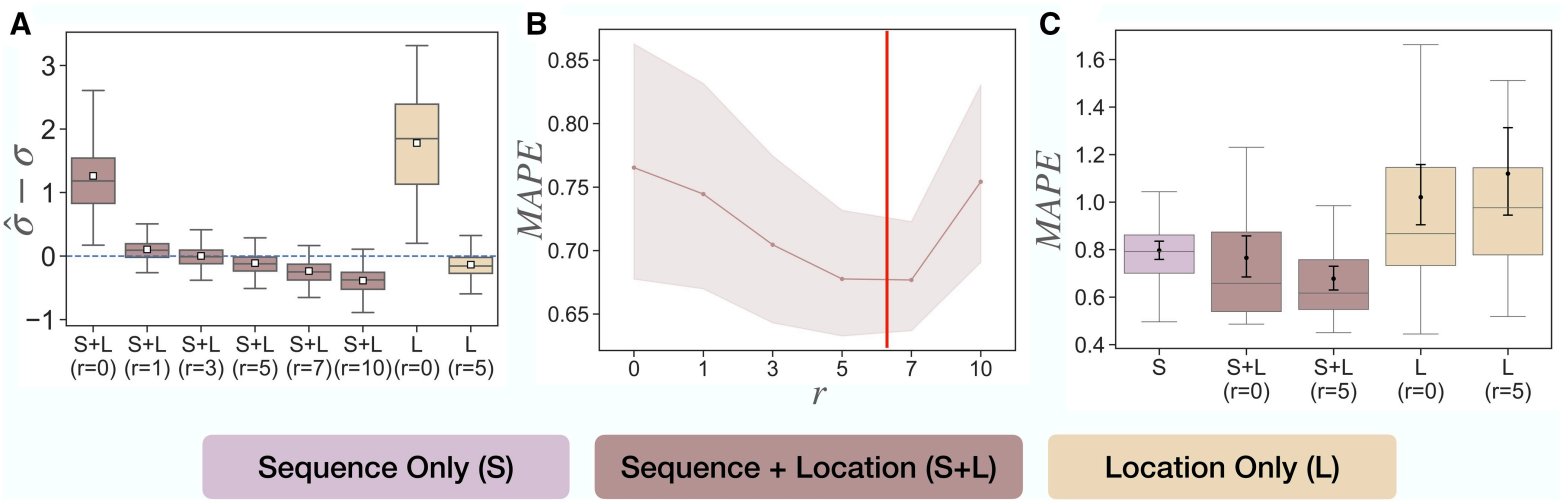
Estimation errors of spatial diffusion rate and branch lengths on the semi-simulated data. Each point is the result on one tree (70 tree topologies, with five simulated sequence replicates for each topology, giving 350 samples). (A) Estimation of $\hat{\sigma }-\sigma$ given different models (Sequence Only (S), Sequence + Location (S + L), and Location Only (L)). (B) Branch length estimation error, measured as the mean absolute percentage error (MAPE) versus *r* when both sequence and location data are used. The area shown is the space between the 95% confidence intervals, the middle line is the mean. The red vertical line indicates the cell radius estimated from frame-to-frame data of 6.68. (C) Branch length estimation error (measured as MAPE) under different models (S, S + L, and L).

When using simulated sequence but real spatial data, we run MOLLUSC on varying values of r∈{0,1,3,5,7,10} and study the impact of *r* on the estimates of *σ* (Fig. 4A) and branch lengths (Fig. 4B). Recall that when *r *=* *0, our model reduces to the Brownian diffusion model, where cell displacement through division is ignored. Our results show that when *r *=* *0, $\hat{\sigma }$ is overestimated on all 70 lineage trees, and this overestimation happens regardless of whether the sequence data is used or not. Note that overestimation is expected, because when *r *=* *0, all the effect of SD through division is ignored and the spatial data is explained solely by Brownian motion, which in turn is parameterized solely by *σ*. In such a setting, *σ* is overestimated to account for both diffusion and displacement. When *r *>* *0, the bias term reduces substantially and remains low ( ≤ 0.110) with all tested r∈[1,5]. With larger values of *r*, however, *σ* is underestimated (i.e. *σ* is slightly underestimated when *r *=* *7 with bias –0.235 and more highly underestimated when *r *=* *10 with bias –0.386).

Examining the effect of *r* on branch length estimation (Fig. 4B), we detect an interesting U-shape structure where branch length error first decreases with *r* but later increases, hinting to an *optimal value* of *r* at between 5 and 7 that minimizes branch length error. As mentioned before, we hypothesize that *r—*which is the displacement magnitude in our model—is also the actual cell radius. To test this hypothesis, we use a provided image of the cells from intMEMOIR to manually detect the locations of cells and measure their radii (see Supplementary Fig. S1). From this study, we get the average radius of the cells to be ∼6.68 and show it in Fig. 4B using the solid red line. This estimated radius is very close to the projected optimal value of *r* that minimizes branch length error, supporting our hypothesis about the connection between displacement magnitude and cell radius.

Next, we test different modalities of MOLLUSC on this dataset, including Sequence Only (S), Location Only (L), and Sequence + Location (S + L) (Fig. 4C). We observe that the error of estimated *σ* and branch lengths are both minimized at *r *=* *5. Therefore, we use *r *=* *5 as the default value of *r* for this data. We observe the same trend as in the fully simulated data: the branch length estimation error of Sequence + Location (S + L) at *r *=* *5 (average MAPE = 0.678) is lower than that of Sequence Only (S) (average MAPE = 0.797); the difference is significant according to ANOVA test with *P*-value $6.6\;\times \;10^{\;-\;4}$. Importantly, we do not gain improvement on branch length error if SD is ignored (i.e. S + L at *r *=* *0), pointing to the role of SD in the model of cell motility. We also note, however, that when we use Location Only, having non-zero *r* sometimes has a negative impact on branch length estimation, possibly due to overfitting (i.e. as mentioned before, the model has many more parameters if r≠0 and requires more data for parameter estimation). Nevertheless, the difference between Location Only with *r *=* *0 and *r *=* *5 is insignificant (according to the ANOVA test, *P*-value .401).

#### 3.2.4 Evaluation on the real intMEMOIR lineage tracing data

We evaluate MOLLUSC on the intMEMOIR lineage tracing data using both the measured mutations (sequence data) and measured cell positions (location data) at the final time. Note that unlike the other settings where the sequence data was simulated under the correct model that we assumed, in this case there can be model misspecification in Sequence data that amplifies the error of parameter estimation.

**Figure 5.**
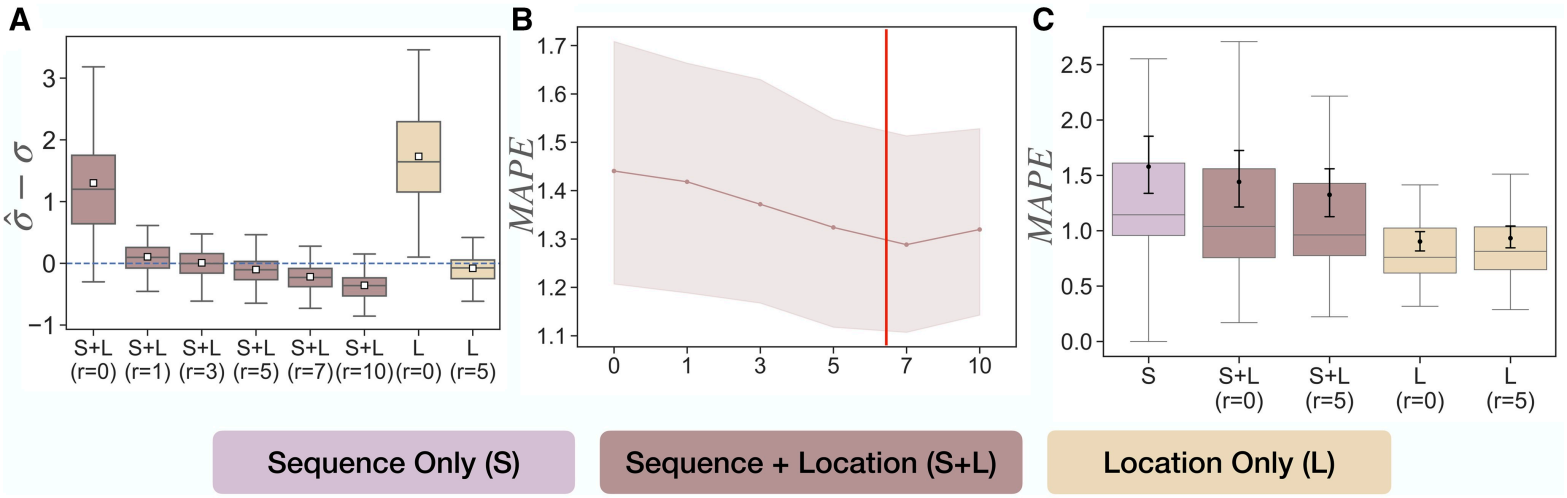
Estimation errors of spatial diffusion rate and branch lengths on the semi-simulated data. Each point is the result on one tree (106 trees). (A) Estimation of $\hat{\sigma }-\sigma$ given different models (Sequence Only (S), Sequence + Location (S + L), and Location Only (L)). (B) Branch length estimation error, measured as the mean absolute percentage error (MAPE) versus *r* when both sequence and location data are used. The area shown is the space between the 95% confidence intervals. (C) Branch length estimation error (measured as MAPE) under different models (S, S + L, and L).

Results on estimation of *σ* and branch lengths (Fig. 5A and B) are consistent with semi-simulated data. That said, if one ignores displacement through division (i.e. *r *=* *0), *σ* will be overestimated regardless of whether the sequence data is used or not (Fig. 5A). There is a good range of *r* around 1 to 5 where the estimate $\hat{\sigma }$ is unbiased, but increasing *r* beyond that leads to underestimation of *σ*. The result on the impact of *r* on branch length error is also consistent with the semi-simulated data. We also detect a U-shape structure in the plot (Fig. 5B), and the optimal value of *r* that minimizes branch length error is still between 5 and 7, very close to the average cell radius of 6.68 estimated from imaging data. On this data, we also examine the accuracy of estimating the mutation rate *λ*, and find that the estimate is always accurate with or without spatial data (see Supplementary Section S5).

As before, we also test different modalities of MOLLUSC on this dataset, including Sequence Only (S), Location Only (L), and Sequence + Location (S + L) (Fig. 5C). There is a subtle difference in branch length error between Sequence Only and Sequence + Location with *r *=* *0, but adjusting *r* to 5 gives a more pronounced improvement, especially on outliers. A unique feature of this real data, however, is the relative performance of Location Only (L) compared to the other two settings where sequence data is incorporated. Interestingly, we observe that solely using location data gives the best branch length estimation. This result shows that not only the location data has more signal for branch length estimation than sequence data, but also the incorporation of sequence data actually has a negative impact. While this fact motivates the usage of location data for lineage tree inference, at the same time it raises concerns about model misspecification in sequence data that can severely degrade the accuracy of lineage inference.

## 4 Discussion

Spatial lineage tracing technologies hold great promise for studying the temporal and spatial components of developmental processes, but are hindered by a lack of reliable computational methods and relatively poor sequence data quality compared to traditional lineage tracing. Here, we propose a novel phylogeographic model—the SD model—for spatial lineage tracing data where we model cell motility as a composite of SD of daughter cells from their parent after cell division, followed by Brownian motion diffusion of individual cells. Combining the SD model with the PMM sequence model described in (Mai *et al.*, 2024), we obtain the first phylogeographic model for spatial lineage tracing. In addition, we develop MOLLUSC, a ML inference framework designed specifically for spatial lineage tracing, where one can combine any spatial model with a sequence model to create a phylogeographic model and perform ML inference.

On both simulated and real spatial lineage tracing data, we demonstrate that the joint model of cell sequence and location gives superior branch length estimation than sequence data alone. These contributions are a first step towards understanding the nuanced interplay between how cellular function is informed by spatial context and ancestry, as well as how these two factors influence each other. The combination of new spatial lineage tracing technologies and novel phylogeographic models will enable deeper insights into the spatiotemporal processes of organismal development.

There are several possible directions for future work. First, the ability to automatically estimate the cell radius *r* instead of requiring it as an input from users is desired. We attempted to jointly estimate *r* with other parameters of the model in the MOLLUSC framework, but found a systematic bias when *r* and *σ* are co-estimated. Specifically, *r* tends to be overestimated while *σ* is underestimated (see Supplementary Section S6), indicating that a more careful treatment is necessary. One approach could be to allow the user to specify a *prior* distribution on *r* and find the maximum a posterior (MAP) estimate. Our results show there is a wide range of *r* (between 1 and 10) that the SD model has higher accuracy compared to the Brownian-only model, thus we speculate that a uniform prior on *r* to constrain *r* to be within a reasonable range would be sufficient for accurate inference. Second, one should try extending the model to other spatial contexts. The intMEMOIR data included cells growing on a dish and thus a Brownian motion was appropriate. However, real tissues and organisms may have other growth patterns that require more specialized spatial models. The literature on cell motility is rich with studies on the effect of chemotaxis (Horwitz and Webb, 2003, SenGupta *et al.*, 2021, Roca-Cusachs *et al.*, 2013), cell adhesion (Huttenlocher *et al.*, 1995), collisions (DiMilla *et al.*, 1991, Vicente-Manzanares *et al.*, 2005), and collective behavior (Friedl and Gilmour, 2009; Rørth, 2009; Weijer, 2009; George *et al.*, 2017), that would be interesting to consider.

Another direction is to develop a better model for sequence data. For example, one could model sequencing error—a missing ingredient in the PMM model (Mai *et al.*, 2024). In addition, the assumptions about a constant mutation rate both through time (i.e. molecular clock) and across target sites (i.e. homogeneous sites) could be relaxed for more accurate estimation. While one can borrow from existing models and methods in phylogenetics literature to relax the clock (Drummond *et al.*, 2006, Ho and Duchêne, 2014, Volz and Frost, 2017, Mai and Mirarab, 2021, Mai *et al.*, 2022) and allow rate heterogeneity (Gu *et al.*, 1995, Mayrose *et al.*, 2005, Stamatakis, 2006), we note that applying these more sophisticated models to the sequence data of spatial lineage tracing that has small number of sites and mutated states can lead to overfitting, though we believe that spatial lineage tracing technologies with higher quality will emerge within the coming years.

Finally, we focus on the branch length and spatial diffusion parameter estimation in this study, however, our proposed ML inference framework can be extended to infer tree topology and locations of ancestral cells. While it is known that ML inference of tree topology is NP-hard (Roch, 2006), one can employ one of the popular heuristic approaches implemented in popular software packages (Minh *et al.*, 2005; Guindon *et al.*, 2010; Price *et al.*, 2010; Stamatakis, 2014) for topology search. ML inference of ancestral locations under the Brownian motion model has been studied before (Lemmon and Lemmon, 2008). We believe such an approach can be generalized to the SD model.

## Supplementary Material

btae221_Supplementary_Data

## Data Availability

All data used in this research is available at https://github.com/raphael-group/intmemoir-processed-data. The MOLLUSC software is available at https://github.com/raphael-group/MOLLUSC.
