# Supplementary material for: Maximum likelihood phylogeographic inference of cell motility and cell division from spatial lineage tracing data
Source: Bioinformatics. 2024 Jun 28;40(Suppl 1):i228–36. doi: 10.1093/bioinformatics/btae221 (PMC11211844; doi:10.1093/bioinformatics/btae221)
Supplement: btae221_Supplementary_Data [file btae221_supplementary_data.zip › btae221_Supplementary_Data/Mai.280.sup.1.pdf]

Maximum Likelihood Phylogeographic Inference of Cell Motility and Cell Division from Spatial Lineage Tracing Data - Supplement

March 21, 2024

Contents

S1 The intMEMOIR real data 2

S2 Simulation details 2
- S2.1 Simulation of the Spatial data . . . 2
  - S2.1.1 Computing the diffusion rate σ from intMEMOIR . . . 2
  - S2.1.2 Computing the cell radius r from intMEMOIR . . . 2
- S2.2 Simulation of the Sequence data . . . 3
- S2.3 Simulation of fully synthetic data for benchmarking . . . 3
- S2.4 Simulations with Additional Sites . . . 4

S3 Assessing the impact of symmetric displacement over time 4

S4 Supplementary method 5
- S4.1 Proof of the multivariate distribution of the observed cell locations . . . 5
- S4.2 Linear-time algorithm for likelihood computation . . . 6

S5 Results of estimating the mutation rate λ 7

S6 Results of estimating the cell radius r 7

S1 The intMEMOIR real data

intMEMOIR is a synthetic lineage recording system recently developed by Chow *et al.* in 2021 [1]. This system utilizes serine integrases to irreversibly edit a multi-state memory DNA array at defined genomic sites upon induction by doxycycline. The editing process occurs stochastically and irreversibly, with any array element susceptible to modification at any time. Chow *et al.* demonstrated the recording and readout capabilities of intMEMOIR in mouse embryonic stem cells, where lineage information was stored and retrieved using microscopy. During experiments, 106 cell colonies were monitored, each originating from an individual cell with an unedited 10-character array. Sequence data was recorded using single molecule fluorescent in situ hybridization (smFISH), while (groundtruth) lineage trees were obtained from time-lapse movies. In addition, cell locations at multiple time points (i.e. 216 time points in total) and some images of the cells are also available.

The intMEMOIR spatial lineage tracing data is publicly available at https://doi.org/10.22002/D1.1444. In this work, we used the following data modules of intMEMOIR: (i) the frame-by-frame recorded cell locations (i.e. the time-lapse movies), (ii) the (groundtruth) lineage trees, (iii) the Sequence data (i.e. character matrices), and (iv) the Imaging data. All are available from intMEMOIR. Refer to the original paper for more details.

S2 Simulation details

In this section we give details about all the simulations used in this work.

S2.1 Simulation of the Spatial data

We used the SD model to simulate Spatial data. In addition to the tree topology and time-resolved branch lengths that are published in intMEMOIR [1], the SD model requires the following additional parameters: the diffusion rate σ, the cell radius r, and the displacement angles $\{\theta_v\}$. To simulate the displacement angles $\{\theta_v\}$, we draw each angle independently from a Uniform distribution $U(0, 2\pi)$. Below we give more details about the selection of σ and r.

S2.1.1 Computing the diffusion rate σ from intMEMOIR

To compute σ we used the frame-by-frame data of intMEMOIR which tracks cell locations at constantly sampled time points. To separate diffusion from division displacement, we filter out frames where cell division occurs. For the remaining time frames, we compute for each cell the movement on x and y axes at each pair of consecutive time frames, and get the two distributions of Δ_x and Δ_y. Using Brownian motion model for cell diffusion at non-dividing time frames and assuming diffusion on x and y coordinates are independent and have the same diffusion rate, the estimate of σ is simply the standard deviation of the combined distribution of Δ_x and Δ_y. This procedure gives an estimate of σ to be 1.5, which we used in all of our simulations. In addition, $\sigma = 1.5$ was used as the groundtruth to evaluate the estimate of σ on the real spatial data.

S2.1.2 Computing the cell radius r from intMEMOIR

We estimated the average radius of cells in our experiments by examining a still movie frame of the int-Memoir experiment, as follows. First, we manually annotated the observed cells (Fig. S1A) in the frame and measured the x and y dimensions of each cell. Then we converted from pixels to the distance units

used in the data and obtained a distribution of the cell radii (Fig. S1B). The average radius r was estimated to be 6.68.

(A) (B)

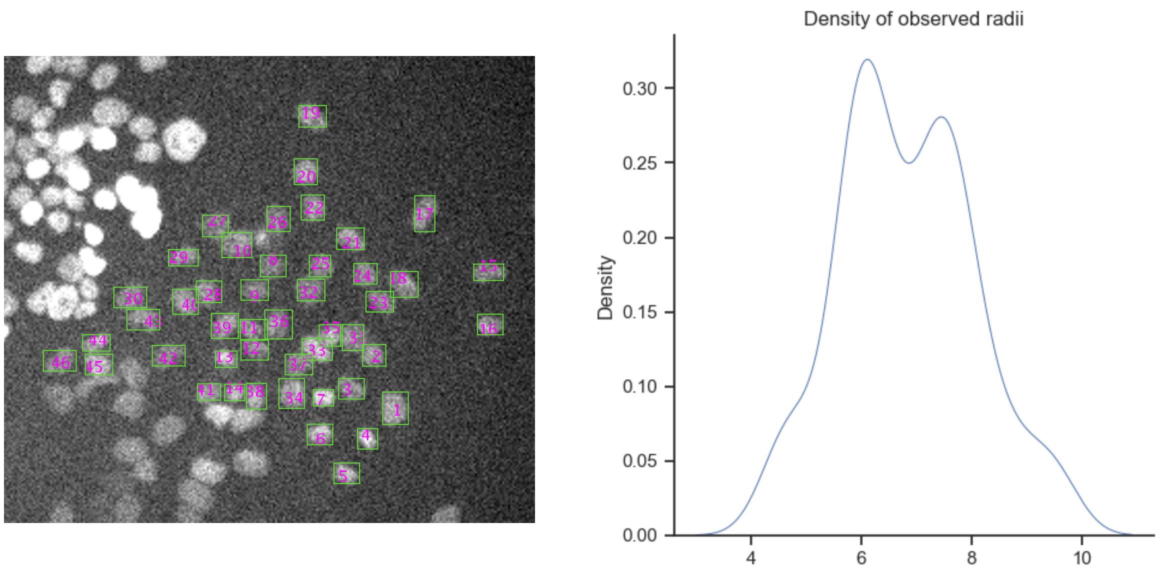

Figure S1: (A) Still image of a video frame from intMemoir. The green boxes are hand annotations to measure the size of each labeled cell. (B) Density plot of observed radii in a single video frame. The x and y radii are considered separate entries for this plot in the case of non-circular cells.

S2.2 Simulation of the Sequence data

To simulate the Sequence data, we selected parameters of the PMM model [3] to match the characteristics of the sequence data of intMEMOIR. In particular, the sequence length K was set to 10 and the alphabet of every site k was set to $\mathscr{A}^{(k)} = \{0, 1, 2\}$, matching those observed in the sequence data of intMEMOIR. Because the intMEMOIR's sequence data does not have missing entries, we set both the missing data rates of PMM to 0 (i.e. $\phi = \nu = 0$).

The mutation rate λ was estimated by fitting the PMM model to the provided sequences, as follows. Let p_0 be the observed proportion of 0 entries in the sequence data. According to the PMM model, $p_0 = e^{-\lambda\tau(1-\nu)} = e^{-\lambda\tau}$, where τ is the length of the experiment (in time unit). Therefore, λ can be estimated as $-\frac{\log p_0}{\tau}$. We compute p_0 by counting the number of zero entries in all samples of intMEMOIR and divide by the combined sizes of all character matrices. The experiment time τ of intMEMOIR is known to be 215 time frames.

S2.3 Simulation of fully synthetic data for benchmarking

While the intMEMOIR dataset has 107 lineage trees, we only used those that have at least 10 leaves, leaving us with 70 samples. Given the properties of real intMEMOIR data, we set $\lambda = 0.006$ and sequence length was set to 10. In the simulated location setting we set $r = 0$ (no division) to serve as a baseline case, allowing for a more complicated spatial patterning of cells to be benchmarked using the real location setting.

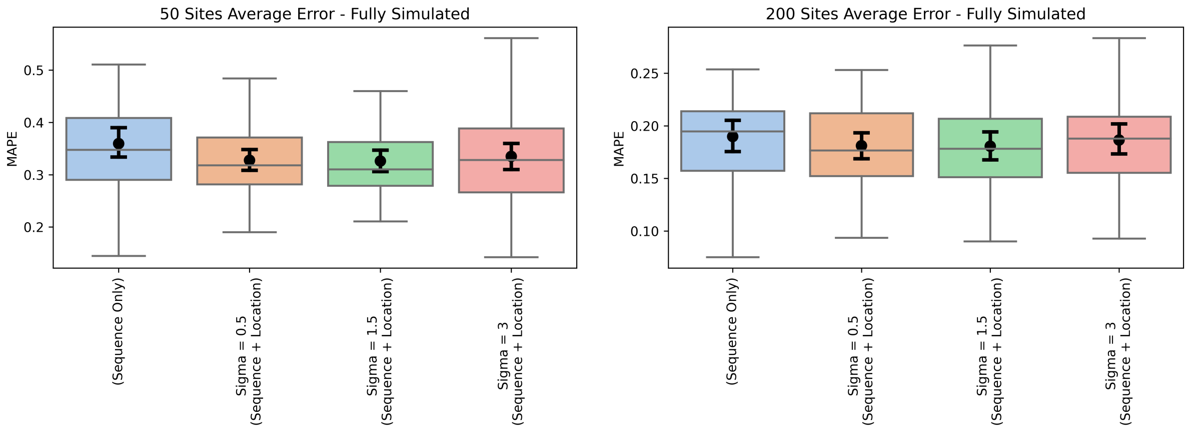

Figure S2: Relative branch length error in a simulation setting of 50 sites (Left) and 200 sites (Right) for the character matrix. Simulations were done on the same locations and tree topologies as the 10 site simulation in the main paper.

S2.4 Simulations with Additional Sites

To demonstrate the relative effect that a more powerful mutation matrix could provide in the branch length estimation process (and to see what kind of benefit spatial information could provide if the mutation matrix was more powerful) we also run simulations where we increase the number of sites. The original intMemoir data uses 10 sites by design – we simulate observed character matrices that use 50 and 200 sites, with the same character constraints (3 states) as the original intMemoir data.

We then test our modal utilizing just these improved charactater matrices, as well as with simulated location data simulated as a normal distributed with different variance amounts.

S3 Assessing the impact of symmetric displacement over time

One question that naturally arises from our symmetric displacement model is under what conditions should be we expect using the symmetric displacement to have a positive impact on modeling branch lengths and/or spatial diffusion rates. As time goes to infinity, symmetric displacement and Brownian motion become essentially indistinguishable. We answer this question in S3 by comparing the relative branch length error to the true branch lengths. All of these trees have have a total height of 215 time units. On real data, we show that including spatial information improves upon sequence-only estimates across time spans, though the benefit of the symmetric displacement model seems to occur mostly when considering the shorter time lengths.

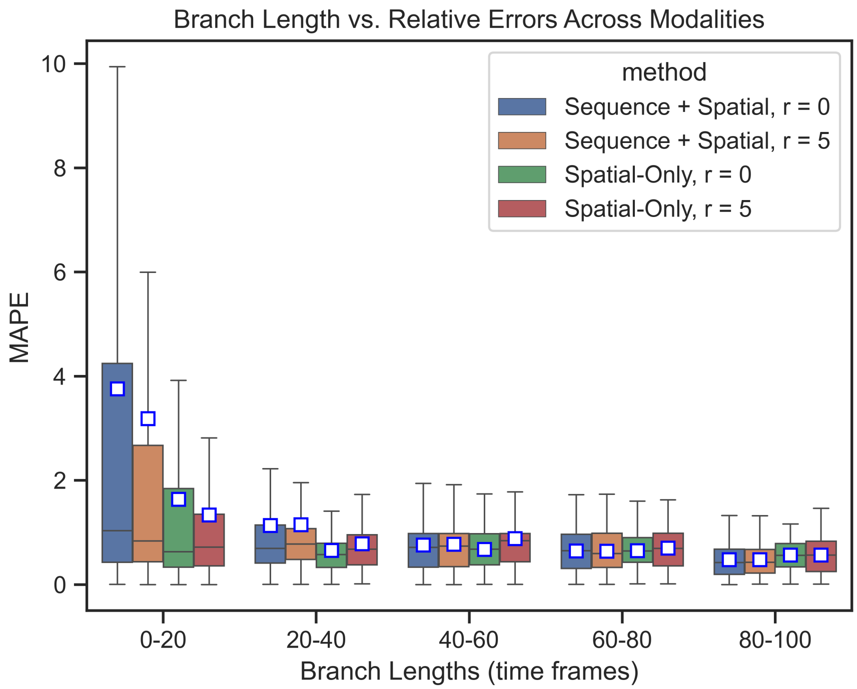

Figure S3: True Branch Lengths vs. Relative Branch Length Reconstructive Error on real intMEMOIR trees n = 106. Branch lengths that didn't fit into these bins (representing 20 time units each) were omitted.)

As expected, the relative branch length error decreases as the number of sites increases. Figure S2 illustrates the relative branch length error under these simulation settings. We note that when using either 50 or 200 sites, the average error still decreases upon incorporating the spatial likelihood, suggesting that the combination of the two still offers a benefit to branch length reconstruction even when the quality of the character matrix is high.

S4 Supplementary method

S4.1 Proof of the multivariate distribution of the observed cell locations

Lemma S4.1.1. *Under the Symmetric Displacement (SD) model, for all leaf nodes w and all pairs of leaf nodes $v \neq w$ of the cell lineage tree T, we have:*

$$x_w \sim N\left(x_0 + \sum_{\substack{(u,v)\in \\ Path(r_T,w)}} s_{u,v} r \cos(\theta_u), \sigma^2 \delta(r_T, w)\right) \tag{S1}$$
$$cov(x_v, x_w) = \sigma^2 \delta(r_T, lca(v, w))$$

where $Path(\cdot,\cdot)$ denotes the path between two nodes, $lca(\cdot,\cdot)$ denotes the least common ancestor of two nodes, and $s_{u,v} = 1$ if v is the left child of u and $s_{u,v} = -1$ otherwise.

Proof. For any leaf node w of T, we have:

$$x_w = \sum_{\substack{(u,v)\in \\ \text{Path}(r_T,w)}} (x_v - x_u) + x_0$$

Recall that under the SD model, for every pair of parent-child (u, v), $(x_v - x_u)$ is normally distributed with mean $s_{u,v} r \cos\theta_u$ and variance $\sigma^2\delta_v$. Therefore, x_w is a sum of normal random variables, and is therefore also normally distributed.

$$\mathbb{E}[x_w] = \sum_{\substack{(u,v)\in \\ \text{Path}(r_T,w)}} \mathbb{E}[x_v - x_u] + x_0 = x_0 + \sum_{\substack{(u,v)\in \\ \text{Path}(r_T,w)}} s_{u,v} r \cos(\theta_u)$$

In addition, because $(x_v - x_u)$ are independent for all $(u, v) \in \text{Path}(r_T, w)$, we have:

$$\text{Var}[x_w] = \sum_{\substack{(u,v)\in \\ \text{Path}(r_T,w)}} \text{Var}[x_v - x_u] = \sigma^2 \sum_{\substack{(u,v)\in \\ \text{Path}(r_T,w)}} \delta_v = \sigma^2\delta(r_T, w)$$

The covariances of every pair of leaves are the same as in the Brownian motion model and have been derived by Felsenstein in [2]. Refer to the original proof for more details. □

S4.2 Linear-time algorithm for likelihood computation

For continuous characters assumed to change according to Brownian motion, Felsenstein's algorithm [2] provides an efficient computation on the likelihood given the tree topology and data at the leaves of the tree. If $r = 0$, then we use Felsenstein's algorithm as described in the original paper for the likelihood of the spatial data, treating x and y coordinate data as two different characters.

As our model has the additional feature of displacement in the cell locations at each division point, we can define the μ_{u_k} (defined as in Felsenstein's paper) recursively, as from our model we are given an expression for the expected values for each of the leaf vertices, and the expectations of the internal nodes are described in a bottom-up fashion. Thus, the computation of the likelihood of a continuous character (from original paper, the node u_k is defined in the original paper as an intermediate node of the pruning process) can still be calculated exactly and with the same procedure (given that we store the needed expected values):

$$[\prod_{\{u_k\}} \frac{1}{\sigma\sqrt{\delta_{u_k}}\sqrt{2\pi}} \exp(\frac{-1}{2}\frac{(u_k - \mu_{u_k})^2}{\sigma^2\delta_{u_k}})][\frac{1}{\sigma\sqrt{\delta_n}\sqrt{2\pi}} \exp(\frac{-1}{2}\frac{(x_n - x_0)^2}{\sigma^2\delta_n})] \tag{S2}$$

We assume the reader is familiar with the original algorithm presented by Felsenstein and thus omit a full formal proof, instead presenting the following observations to show that it is possible to use this equation with dynamic programming.

Observation S4.2.1. *The likelihood of the tree at the beginning of each while loop iteration is equal to the likelihood of the pruned tree at the end of the iteration times a normal likelihood with mean equal to μ_{u_k} and variance equal to $\sigma^2 b_{u_k}$ where k is the node label of the immediate ancestor of the two pruned leaf nodes.*

This follows from the fact that our algorithm doesn't change the values of the variances or covariances compared to the original likelihood, and thus the same reasoning from the base Brownian motion for why the pruning operation works still applies.

Observation S4.2.2. *Each value of μ_{u_k} for all values of k representing the internal nodes of the tree, can be expressed only as a combination of the displacement terms, and not x_0*

For the sake of simplicity, when describing the value of a linear combination of the displacement terms (for example, the sum term of the μ_w of the initial leaves of the tree, we replace the linear combination of displacement terms with the term `displace`, which represents a value that can be expressed solely as

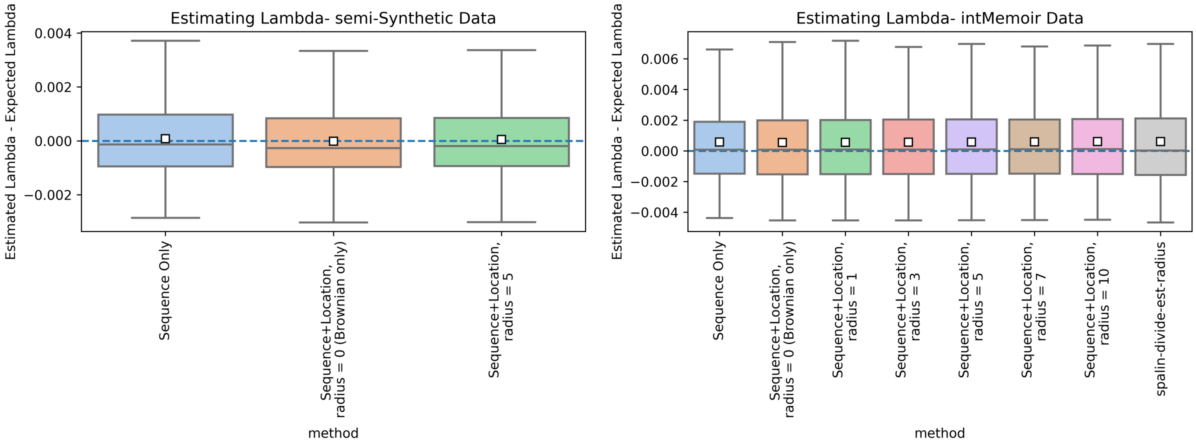

Figure S4: Estimated mutation rate λ on semi-synthetic data (Left) and real intMEMOIR data (Right).

a linear combination of the displacement terms. Thus to describe $\mu_w = x_0 + \sum_{v \in \mathcal{P}(r_T, w)} \chi_v$, we write: $\mu_w = x_0 + \mathtt{displace_w}$

Observe that initially, $E[x_w] = x_0 + \mathtt{displace_w}\ \forall w \in \mathscr{L}_T$. When we prune the tree by letting $u_k = x_i - x_j$, and $x_k = \frac{\delta_j}{\delta_i + \delta_j} x_i + \frac{\delta_i}{\delta_i + \delta_j} x_j$, the following is true:

1. $E[u_k] = E[x_i] - E[x_j] = [x_0 + \mathtt{displace_i}] - [x_0 + \mathtt{displace_j}] = \mathtt{displace_i} - \mathtt{displace_j}$
2. $E[x_k] = \frac{\delta_j}{\delta_i + \delta_j} E[x_i] + \frac{\delta_i}{\delta_i + \delta_j} E[x_j] = \frac{(\delta_i)(x_0 + \mathtt{displace_i}) + (\delta_j)(x_0 + \mathtt{displace_j})}{\delta_i + \delta_j} = x_0 + \frac{(\delta_j)(\mathtt{displace_i}) + (\delta_i)(\mathtt{displace_j})}{\delta_i + \delta_j} = x_0 + \mathtt{displace_k}$

From the two above, we observe that after one iteration of pruning, the μ_{u_k} value can be written as only a displace term, and the value of μ_{x_k} can be written as the sum of x_0 and a displace term. As the x_k value is the one that remains on the tree as a leaf node while the u_k term is pruned away, we still have the property always that for all leaf nodes on the tree after any amount of iterations of pruning $E[x_w] = x_0 + \mathtt{displace_w}\ \forall w \in \mathscr{L}_T$ and thus every iteration will have the above two properties of the expected values of the new replacement values be true.

Note: The original paper [2], drops the last term $[\frac{1}{\sigma\sqrt{b_n}\sqrt{2\pi}} \exp(\frac{-1}{2} \frac{(x_n - x_0)^2}{\sigma^2 b_n})]$ from the likelihood (S2) to turn this into a marginalization. Felsenstein presents several reasons why this is a justifiable procedure, and we defer to the reasons in the original paper.

S5 Results of estimating the mutation rate λ

We illustrate the results of our method on estimating the mutation rate on real data here in Figure S4. The true mutation rate λ was estimated to be 0.006, which we find all modeling and parameter choices to center on without much noticeable difference between the different models.

S6 Results of estimating the cell radius r

We illustrate the results of our method on including the length of the radius as a parameter to estimate in our model in real data. As can be seen in Fig. S5, the radius length tends to overestimated and the sigma amount tends to be underestimated when letting our model estimate the radius as another parameter.

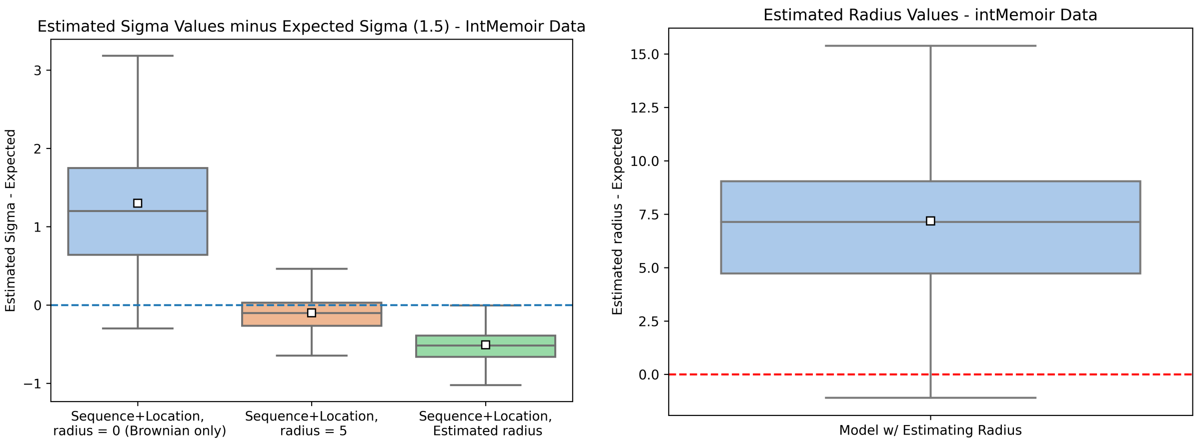

Figure S5: Estimated Sigma (Left) and radius length (Right) on Real IntMemoir Data. The red line shows the expected radius estimated from imaging data subtracted from the estimated radius.

References

[1] K.-H. K. Chow, M. W. Budde, A. A. Granados, and et. al. Imaging cell lineage with a synthetic digital recording system. *Science*, 372(6538):eabb3099, 2021.

[2] J. Felsenstein. Maximum likelihood and minimum-steps methods for estimating evolutionary trees from data on discrete characters. *Systematic Zoology*, 22(3):240, Sept. 1973.

[3] U. Mai, G. Chu, and B. Raphael. Maximum likelihood inference of time-scaled cell lineage trees with mixed-type missing data. *bioRxiv and RECOMB (in prep)*, 2024.

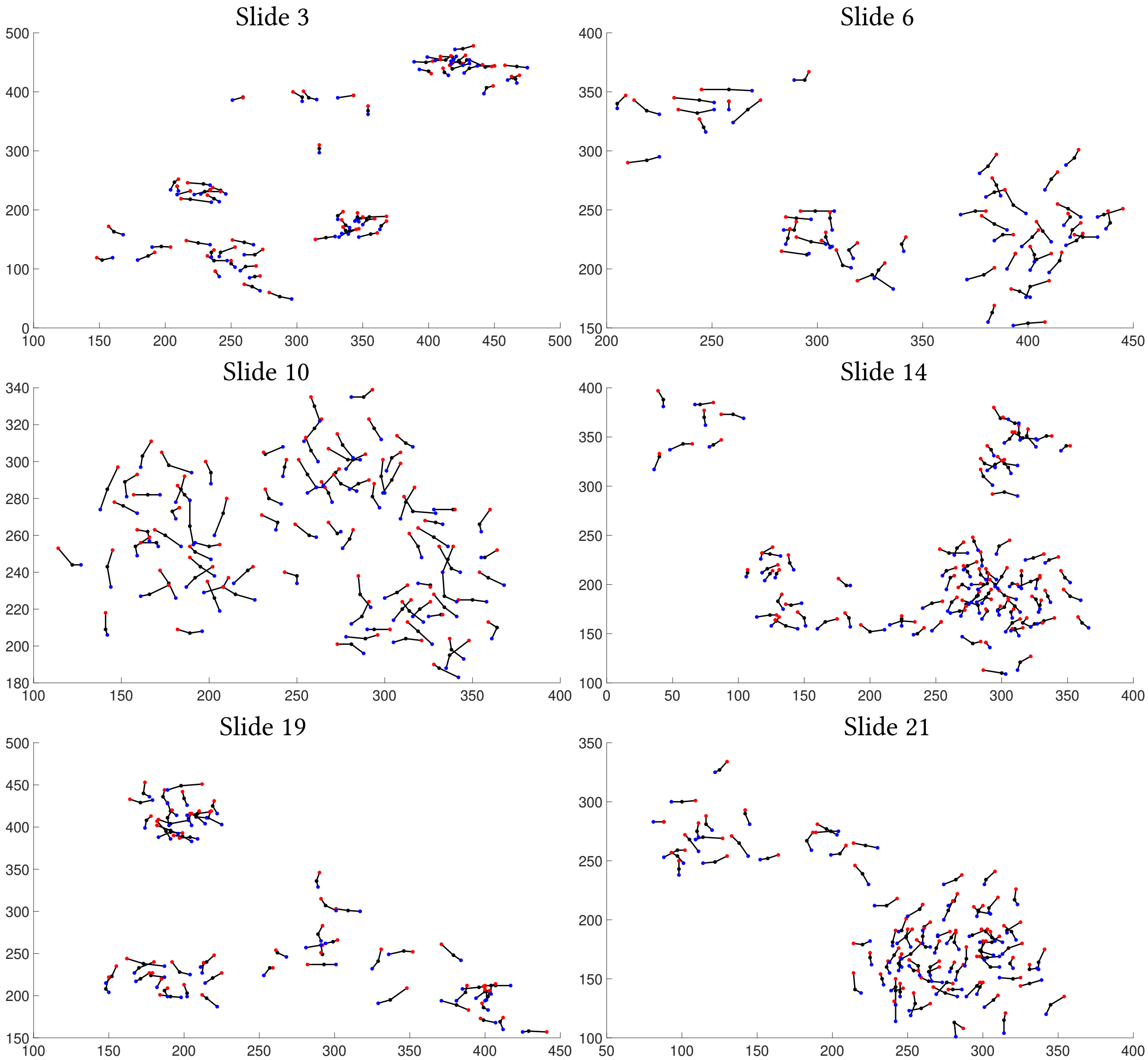

Figure S6: Empirical locations of the parent cell (shown in black) and the two daughter cells (shown in blue and red) at the division frames of the intMEMOIR data (combined of all cells of each slide).
